# Murine Non-Transgenic Models of Alzheimer’s Disease Pathology: Focus on Risk Factors

**DOI:** 10.3390/brainsci15030322

**Published:** 2025-03-19

**Authors:** Maricarmen Hernández-Rodríguez, Juan Manuel Vega López, Martín Martínez-Rosas, María Inés Nicolás-Vázquez, Elvia Mera Jiménez

**Affiliations:** 1 Laboratorio de Cultivo Celular, Neurofarmacología y Conducta, Escuela Superior de Medicina, Instituto Politécnico Nacional, Plan de San Luis y Díaz Mirón s/n, Mexico City 11340, Mexico; elviamj@gmail.com; 2 Departamento de Química Inórgánica, Escuela Nacional de Ciencias Biológicas, Prolongación de Carpio y Plan de Ayala s/n, Mexico City 11340, Mexico; vegaj5648@gmail.com; 3 Departamento de Fisiología, Instituto Nacional de Cardiología Ignacio Chávez, Juan Badiano 1, Mexico City 14080, Mexico; martin5163@hotmail.com; 4 Departamento de Ciencias Químicas, Facultad de Estudios Superiores Cuautitlán, Universidad Nacional Autónoma de México, Cuautitlán Izcalli 54740, Mexico; nicovain@yahoo.com.mx

**Keywords:** Alzheimer disease, non-transgenic models, amyloid β

## Abstract

Alzheimer’s disease (AD) represents a significant challenge among neurodegenerative disorders, as effective treatments and therapies remain largely undeveloped. Despite extensive research efforts employing various methodologies and diverse genetic models focused on amyloid-β (Aβ) pathology, the research for effective therapeutic strategies remains inconclusive. The key pathological features of AD include Aβ senile plaques, neurofibrillary tangles (NFTs), and the activation of neuroinflammatory pathways. Presently, investigations into AD and assessing potential treatments predominantly utilize Aβ transgenic models. Conversely, non-transgenic models may provide valuable insights into the multifaceted pathological states associated with AD. Thus, these models may serve as practical complementary tools for evaluating therapeutic and intervention strategies, since the primary AD risk factors are most frequently modeled. This review aims to critically assess the existing literature on AD non-transgenic models induced by streptozotocin, scopolamine, aging, mechanical stress, metals, and dietary patterns to enhance their application in AD research.

## 1. Introduction

Alzheimer’s disease (AD) is the most common type of dementia worldwide and accounts for up to 70% of all dementia diagnoses [1]. Its incidence escalates significantly with age, and AD is classically classified into two primary forms: sporadic AD (SAD) (or late-onset AD) and familial AD (FAD) (or early-onset AD) [2]. SAD constitutes the majority of AD cases (over 90%), and it typically manifests after the age of 65. SAD represents a multifactorial disorder in which both environmental factors and genetic predisposition could contribute to the genesis of the disease [2]. Many risk factors have been linked to the development of SAD, including genetic predisposition, aging, exposure to metals, head injury, diet, mitochondrial dysfunction, vascular disease, immune system dysfunction, infectious disease, and genetic predisposition [3].

In comparison, FAD accounts for less than 10% of AD cases, and it presents before the age of 65, often in the fourth or fifth decade of life. It is caused by autosomal dominant genetic mutations in the Amyloid β Precursor Protein (APP), Presenilin 1 (PSEN1), or Presenilin 2 (PSEN2) genes, and it exhibits a clear inheritance pattern [4].

Great efforts have been invested in understanding AD physiopathology, with early animal experiments playing a crucial role. The evolution of animal models reflects advancements in AD genetics and pathology. Initially, research focused on observing cognitive decline in aging animals. Before the modern era of genetic engineering, researchers relied on naturally aging animals, such as dogs and primates, to study age-related cognitive changes [5,6]. These studies provided initial insights into the behavioral and neuropathological aspects of brain aging. The development of transgenic rodent models marked a significant turning point [7]. Researchers began creating mice that expressed human genes linked to AD, allowing the study of specific pathological features like amyloid plaques and tau protein pathology [8].

In this sense, murine animal models of AD have been established to resemble key histopathological and cognitive impairments [9]. Although many transgenic AD models have been developed to study pathological changes and novel innovative treatments, they are based mainly on FAD. Transgenic models express a mutated form of human APP in addition to a series of mutations in the enzymes that process APP [10]. Thus, the modifications mentioned result in the release of different isoforms of amyloid β (Aβ) peptide [11]. Therefore, the genetic backgrounds of transgenic mice may confound crucial aspects of AD that might not relate to sporadic forms of the disease.

Although FAD shares histopathological characteristics with SAD, including the increase in the deposition of Aβ peptides and the hyperphosphorylation of tau protein in the brains of AD patients [12], most AD cases belong to SAD.

The mechanisms driving the establishment of SAD are more complex and varied than the dominant monogenic mutations that cause FAD [13]. For this reason, non-transgenic models related to SAD pathological changes, which mimic the risk factors, and the pathways involved in the pathogenesis of SAD, could increase the comprehension of the disease and the rate of success during the development and evaluation of novel compounds. In this review, we discuss the key characteristics of established non-transgenic murine models of AD concerning the primary risk factors. We also explore how pharmacologically induced rat models can help address the challenges faced in AD research and drug development.

## 2. Methods

A systematic review was conducted on published studies focused on creating non-transgenic animal models of AD related to the main risk factors. This review covered the pathological changes associated with AD and factors related to SAD. It provided a detailed explanation of non-transgenic animal models of AD, including models induced by streptozotocin, scopolamine, aging, mechanical stress, metals, and dietary patterns. Finally, the advantages and disadvantages of transgenic and non-transgenic AD models are discussed.

## 3. Results

### 3.1. Evolution of AD Definition

The initial definition of AD was based on clinical–pathological criteria [9]. The diagnosis in the living patient was considered probable AD and then confirmed at autopsy when characteristic neuropathological changes were found [14]. Over time, the concept became confusing, as two nosological entities of a different nature were described: a prototypical clinical syndrome without neuropathological verification and, on the other hand, the histopathological findings that confirmed the diagnosis. In the search for a more integrative definition, the National Institute on Aging and the Alzheimer’s Association proposed the following: “AD refers to deposits of pathological Aβ and tau plaques, defined in vivo by abnormal biomarkers of Aβ and pathological tau (both criteria are necessary)”. This definition includes the underlying pathological processes confirmed by post-mortem examination and the in vivo clinical manifestation evaluated by biomarkers [15]. This definition allows a greater understanding of the mechanisms and develops interventions that prevent or delay the initial onset of symptoms of the preclinical phase of AD.

### 3.2. Pathological Changes in AD

AD is characterized by alterations in learning and memory that are correlated with degeneration of several brain areas, including the hippocampus and cerebral cortex. Both FAD and SAD share histopathological characteristics, such as senile plaques composed principally of Aβ, neurofibrillary tangles (NFTs), and neuronal and synaptic loss [16].

#### 3.2.1. Aβ and Its Pathological Forms

**Figure 1:**
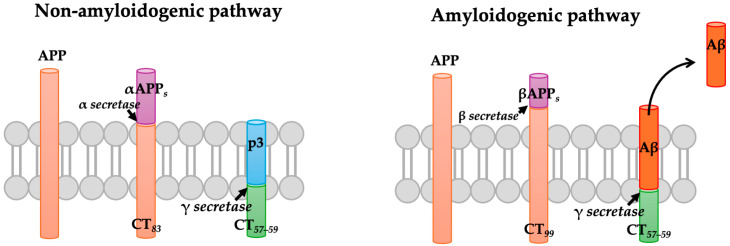
The amyloid pathway. The amyloid precursor protein (APP) can be cleaved through two pathways. In the non-amyloidogenic pathway, APP is first cleaved by α-secretase and then by γ-secretase, resulting in the formation of a non-toxic fragment. In the amyloidogenic pathway, APP is cleaved by β-secretase, followed by γ-secretase, which ultimately leads to the formation of a neurotoxic Aβ peptide that tends to aggregate.

Aβ is a 4-kDa peptide produced by the proteolytic processing of APP [17]. APP is a transmembrane glycoprotein processed by the action of several proteases, following two processes that compete for the same part of the protein [18]. In the most common pathway, a protease known as α-secretase cleaves APP, releasing a soluble, extracellular fragment of about 695 amino acid residues. Then, the rest of the protein which is integrated into the membrane is processed by the γ-secretase, resulting in the release of the carboxyl terminal, which is transported into lysosomal vesicles for further degradation. This process occurs mainly in the healthy brain and is known as the non-amyloidogenic pathway because the action of α-secretase prevents the formation of the Aβ peptide (Figure 1) [19]. However, in the brains of AD patients, most of the APP is processed differently, following the amyloidogenic pathway. In this pathway, the APP is cleaved firstly by β-secretase (BACE1), resulting in the release of a longer carboxylic fragment of 99 amino acids, which is subsequently processed by the γ-secretase, resulting in the release of Aβ peptide (Figure 1). Once Aβ is released by the sequential action of β and γ secretases, it interacts with other monomers, resulting in the formation of soluble oligomers (Aβos) and insoluble fibrils (Aβfs) [20].

Senile plaques are composed principally of a central core of insoluble Aβf surrounded by abnormally formed neurites. Additionally, it is possible to find microglial cells and, less frequently, reactive astrocytes in the periphery of senile plaque.

In addition, Aβ can be found as focal diffuse deposits in diverse brain areas, including the cerebral cortex. Interestingly, these deposits are not accompanied by dystrophic neurites or surrounding activated glial cells. Diffuse plaques are not exclusive to AD; in elderly individuals, they can be seen in relatively large numbers of diffuse plaques in the brains of patients without cognitive impairment [21]. In addition, Aβ deposits can be detected in the walls of arterial vessels in the meninges and the cerebral cortex. When this kind of Aβ deposit is minimal, it does not impair the function of the vessels; however, when the deposits severely affect the vessels, there is a higher risk for spontaneous vascular rupture, resulting in localized blood accumulation in brain tissue.

In contrast, oAβ is generally considered the most neurotoxic and pathogenic form of Aβ [22]. It has been demonstrated that oAβ accumulation occurs early in AD development, including before plaque formation [23]. The consequence of increased oAβ production in the brain has been widely demonstrated in behavioral, neuropathological, and cell-biological studies [24].

Recent studies have shown that oAβ is capable of binding to various surface receptors, including advanced glycation product receptor (RAGE), α7-nicotinic acetylcholine receptor (α7 nAChR), *N*-methyl-d-aspartate receptor (NMDAR), and cellular prion protein (PrPc), among others, which are highly expressed in neurons and glial cells [25]. oAβ binding to RAGE promotes the development of neuronal and microglial toxicity by several pathways, including the development of oxidative stress, synaptic dysfunction, and, ultimately, neuronal cell death [26]. Indeed, it is shown that RAGE expression is significantly increased in the brain of AD patients, especially in the blood vessels [25]. In addition, oAβ interacts with NMDARs at the postsynaptic terminal, and their activation induces Ca^2+^ dysregulation, neuronal death, and synaptic dysfunction. Nevertheless, it remains uncertain whether oAβ directly interacts with NMDAR subunits [27]. The α7 nAChR is a potential receptor that binds to fAβ with high affinity. This receptor mediates the phosphorylation of tau proteins induced by Aβ through the activation of ERK and JNK signaling pathways. Additionally, Aβ can directly interact with cell membranes, forming ion channels or pores that lead to membrane disruption and, consequently, neuronal damage [28].

In contrast, fAβ induces neurotoxicity and dysfunction of synapses in AD brains by generating reactive oxygen species (ROS). ROS generation increases Aβ levels and promotes its deposit [29]. Interaction of fAβ with with Cu^2+^ ions triggers the production of ROS, which are favored by the high concentration of Cu^2+^ ions in the amyloid plaque deposits in the brains of individuals with AD. Once bound to fAβ, Cu^2+^ ions can directly convert physiological H_2_O_2_ to the hydroxyl radical (·OH). Several experiments proved that when the Cu^2+^ was reduced to Cu^1+^ by biological lowering agents such as vitamin C, cholesterol, and catecholamines, the Cu^1+^-Aβ complex generated ROS [30].

#### 3.2.2. Phosphorylated Tau and NFTs

Tau is a microtubule-associated protein mainly distributed in the axon of the neurons of the healthy brain [31]. Tau regulates the enzymatic activity of phosphatases and kinases through signal transduction mechanisms [32]. Nevertheless, mutations in tau protein result in changes in its affinity to microtubules, changes in its conformation, alterations in posttranslational modifications, and increased aggregation propensity [33]. The pathologic tau changes in AD brains, including phosphorylation and increased aggregating ability, are related to brain damage [34]. In AD, hyperphosphorylated tau is associated with the formation of NFTs, which is related to conformational changes in the structure of tau [35]. Due to modifications in their structure, tau protein is redistributed in both the soma and dendrites of neurons [36]. Recent studies have also shown that tau is present in glial cells. The dynamics of tau and other microtubule-binding proteins, which attach to microtubules, are strictly regulated by various factors to ensure proper function [37]. When tau detaches from microtubules, it accumulates in neurites and neuronal cell bodies, leading to the formation of NFTs, which are significant pathological features of AD. The accumulation of paired helical filaments and NFTs disrupts normal physiological functions, resulting in apoptosis and neuronal loss [38]. This deterioration manifests as cognitive impairment and disease progression, ultimately leading to death. Additionally, mutations in the tau gene are linked to frontotemporal dementia and parkinsonism, indicating that tau dysfunction may play a role in neurodegeneration [39].

### 3.3. Factors Related to SAD

**Figure 2:**
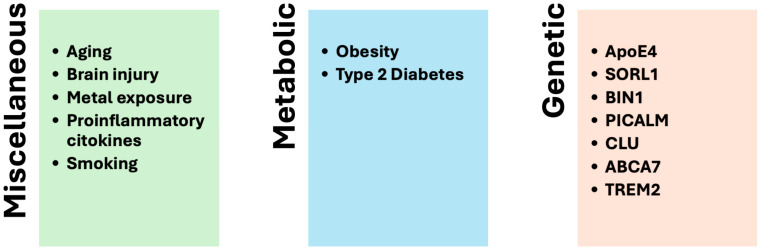
Risk factors involved in the development of SAD.

Research increasingly indicates that in SAD, various risk factors stemming from the interactions between genes and environmental influences contribute to the activation and progression of the disease (Figure 2), particularly in the context of an aging brain [40]. Many researchers in the field of AD suggest that these environmental risk factors play a significant role during the preclinical phase of the illness, which can occur decades before the onset of clinical dementia. Key modifiable risk factors for SAD primarily include cardiovascular issues such as diabetes and obesity, as well as lifestyle habits like smoking, physical activity, diet, and alcohol consumption.

#### 3.3.1. Aging

Aging is the primary and undisputed risk factor for AD [41]. Several critical processes related to aging—such as oxidative stress, mitochondrial dysfunction, impaired blood–brain barrier (BBB) function, and arteriolosclerosis, which can lead to hypoxia—have all been linked to an increased risk of developing AD. Notably, many of these processes are associated with enhanced amyloidogenic processing of APP and the aggregation of Aβ [42].

Research involving AD transgenic mice has shown that cerebral hypoxia increases the activity of BACE1. This enzyme’s activity is regulated by the hypoxia-inducible factor 1-alpha (HIF-1α). Consequently, the increase in BACE1 activity leads to elevated production of Aβ.

#### 3.3.2. Metal Exposure

There is substantial evidence linking metal exposure to the development of AD. An increased metal concentration in the brain is associated with Aβ deposition in senile plaques, leading to a state of oxidative stress that results in neuronal damage [43]. Aβ catalyzes the reduction of Cu^2+^ and Fe^3+^, and in the absence of sufficient antioxidant mechanisms, this process can produce toxic ROS that may contribute to the pathogenesis of AD. The generation of ROS accelerates the aggregation of Aβ by creating species that are less effectively eliminated through the BBB and are more prone to aggregation [44].

AD has been associated with exposure to certain metals, including aluminum (Al^3+^). This metal can enter the body through occupational exposure or by consuming food or water contaminated with traces of aluminum, mainly when using aluminum cookware [45]. Once in the body, aluminum can disrupt the BBB and accumulate in the brain, altering mitochondrial function, depleting adenosine triphosphate (ATP), and producing ROS. This process can lead to cell death through apoptotic or necrotic mechanisms [46]. Additionally, aluminum can inhibit the activity of antioxidant enzymes, disrupting brain neurochemistry and causing oxidative damage to brain DNA.

#### 3.3.3. Brain Injury

Vascular brain injury that leads to reduced blood flow, known as hypoperfusion, can initiate and promote the development of amyloid pathology in the brain. Multiple pathological processes connect traumatic brain injury (TBI) with neurodegeneration and dementia [47]. The term chronic traumatic encephalopathy (CTE) refers to the neurological changes that can occur later in life in individuals who have experienced repeated concussive head injuries. This condition can present symptoms and pathological features similar to those of other neurological diseases, including AD, Parkinson’s disease (PD), frontotemporal dementia, and amyotrophic lateral sclerosis [48]. Postmortem examinations of brains affected by TBI and CTE have revealed pathological features typically associated with AD [49], such as increases in hyperphosphorylated tau (P-tau) and, in certain cases, Aβ deposits. Recent consensus criteria classify CTE as a tauopathy [50].

#### 3.3.4. Proinflammatory Cytokines

In AD patients, the brain exhibits a significant inflammatory response primarily driven by activated microglia and astrocytes [51]. Both species, oAβ and fAβ, stimulate the activation of microglia in the AD brain, leading to a proinflammatory response mediated by cytokines such as interleukin-1 (IL-1), interleukin-6 (IL-6), and tumor necrosis factor-alpha (TNF-α), in addition to the complement system and chemokines. This inflammatory response plays a critical role in the neurodegeneration associated with AD [52,53,54].

Furthermore, elevated levels of circulating proinflammatory cytokines have consistently been reported in individuals with AD [55]. Research has shown that higher serum levels of IL-6, TNF-α, and IL-1 are linked to an increased risk of developing AD. Studies have also identified communication pathways between the peripheral immune and central nervous system, indicating that elevated levels of peripheral proinflammatory cytokines can lead to increased cytokine production in the central nervous system [56].

In AD pathogenesis, central proinflammatory cytokines have been shown to promote the phosphorylation of tau protein and the production of amyloid beta, thereby exacerbating the neurodegenerative process [57].

#### 3.3.5. Type 2 Diabetes Mellitus

Large-scale, population-based prospective studies have demonstrated that type 2 diabetes increases the risk of developing AD [58]. While there is ongoing debate between epidemiological findings and neuropathological observations regarding the relationship between type 2 diabetes and AD, experimental research has linked insulin and insulin resistance to the pathogenesis of AD in several ways. In the brain, insulin and insulin-like growth factor signaling regulate a wide range of functions, including glucose and energy metabolism, as well as the growth, differentiation, migration, survival, and plasticity of neurons [59].

Specifically, in terms of Aβ metabolism, insulin aids in the trafficking of APP and Aβ from the trans-Golgi network to the plasma membrane, which facilitates the extracellular release of Aβ. Additionally, insulin resistance in mice—induced by a high-fat diet—results in increased activity of BACE1 and γ-secretase, contributing to the accumulation of Aβ [60].

#### 3.3.6. Obesity

The human brain contains a significant amount of cholesterol, primarily located in myelin and the plasma membranes of glial cells and neurons [61]. Within cell membranes, cholesterol is associated with specialized lipid microdomains known as lipid rafts. These lipid rafts play a crucial role in the amyloidogenic processing of APP and the oligomerization of Aβ peptide, suggesting that cholesterol may influence these processes [62].

Research involving animal models has indicated that a cholesterol-rich diet tends to increase Aβ accumulation in the brain. This phenomenon can be explained by previous studies demonstrating that cholesterol affects the processing of APP. In cultures of rat hippocampal neurons, lowering cellular cholesterol levels—either by inhibiting de novo synthesis with statins or using the cholesterol-extracting agent methyl-β-cyclodextrin—resulted in a significant decrease in Aβ production and secretion. Furthermore, cholesterol depletion led to a marked reduction in Aβ levels. In contrast, the secretion of the APP ectodomain (produced through the non-amyloidogenic α-secretase pathway) was observed to increase [63].

#### 3.3.7. Smoking

Observational studies indicate a link between current smoking and an increased risk of dementia, SAD, and cognitive decline. It is estimated that nearly 14% of AD cases may be potentially attributable to smoking due to its high prevalence [64]. Smoking may raise the risk of AD through several mechanisms, primarily related to oxidative stress and inflammatory responses [65]. Furthermore, the risk increases significantly with greater cumulative cigarette exposure.

Research has shown that mice exposed to daily cigarette smoke for 15 weeks experience oxidative stress in brain tissue, as evidenced by elevated levels of malondialdehyde, which is a marker of lipoperoxidation [66]. Another study found that rats exposed to daily cigarette smoke for approximately 8 weeks exhibited significantly increased levels of 8-hydroxydeoxyguanosine, a marker of oxidative damage to RNA and DNA nucleosides, particularly in the dentate gyrus and CA3 subfields of the hippocampus [67].

Additionally, Khanna et al. reported that rats exposed to cigarette smoke five days a week for six weeks showed significantly elevated levels of proinflammatory cytokines, including IFN-γ and TNF-α, in the brain. Multiple cytokine genes, such as TNF-α, IL-1α, IL-1β, and Th17, were also upregulated. These changes contribute to the development of neuroinflammation and brain damage [68].

#### 3.3.8. Diet

Diet has been shown to influence the immune system, and various nutrients and bioactive components can affect neuroinflammatory processes in animals [69]. For example, polyphenols, unsaturated fats, and antioxidant vitamins help reduce oxidative stress and neuroinflammation, whereas saturated fats promote inflammation, particularly in the hypothalamus [70].

However, it remains unclear whether the effects of diet on neurocognition are directly mediated by neuroinflammatory processes or by other immune mechanisms in vivo. An increasing amount of evidence suggests that peripheral inflammation and changes in the gut microbiome can enhance neuroinflammation and accelerate neurodegeneration, and these external factors can also be influenced by diet [71].

#### 3.3.9. Alcohol Consumption

Research indicates a connection between the development of cognitive impairment, dementia, and harmful alcohol use [72]. However, the relationship between alcohol consumption and cognitive outcomes is still debated. Observational studies suggest that heavy alcohol consumption is linked to alcoholic dementia and can result in a decline in mental and executive functioning. On the other hand, moderate alcohol intake may offer a protective effect. This relationship between alcohol consumption and cognitive decline is often described as U-shaped [73].

#### 3.3.10. Genetics

FAD is typically characterized by a rapid rate of progression and follows a Mendelian pattern of inheritance. Three main genes—APP, PSEN1, and PSEN2—encode proteins that are involved in the breakdown of APP and the production of Aβ, and they have been strongly linked to the pathophysiology of FAD. In contrast, the genes associated with SAD increase the risk of the disease in a non-Mendelian manner. First-degree relatives of individuals with SAD have a lifetime risk of developing the disease that is twice as high as that of individuals without an affected first-degree relative. Furthermore, SAD is more common in monozygotic (identical) twins than in dizygotic (fraternal) twins, indicating a significant genetic contribution, estimated to be between 60% and 80% [74].

For over a decade, the only well-established genetic risk factor for SAD has been the APOEe4 allele, which is located on chromosome 19q13. This allele is a part of the APOE gene, which encodes a lipid-binding protein and has three common isoforms: APOEe2, e3, and e4. Having a single APOE-e4 allele is associated with a 2- to 3-fold increased risk of developing AD, while having two copies can increase the risk by 5-fold or more [74]. Additionally, APOEe4 is linked to lower cognitive performance, particularly in the memory domain, and is associated with mild cognitive impairment (MCI) and the progression from MCI to dementia [75].

In addition, genome-wide association studies have identified polymorphisms in or near several genes that are associated with AD risk, including ABCA7, CLU, SORL1, BIN1, PICALM, and TREM2 [76].

### 3.4. Non-Transgenic Models

**Figure 3:**
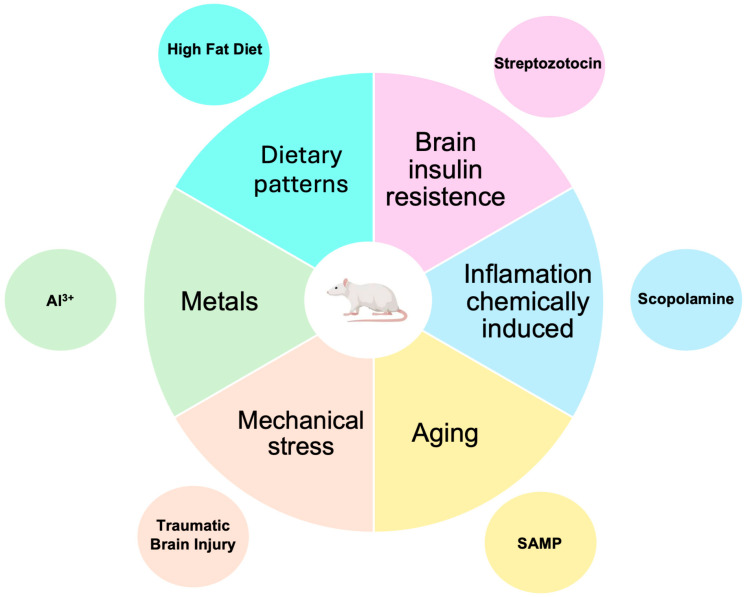
Non-transgenic murine models for AD research. Although these models employ mechanisms related to risk factors for developing SAD (which accounts for 95% of AD cases), they are less commonly used than transgenic models.

Considering the many factors related to SAD, non-transgenic mouse models have been developed and used to evaluate new treatments, as shown in Figure 3. However, these models are less well-known and employed than transgenic models. As Figure 3 shows, these models are developed by mimicking the risk factors for SAD.

#### 3.4.1. Models Induced by the Administration of Streptozotocin (STZ)

Streptozotocin (STZ) is a bacterial toxin that selectively damages pancreatic β cells. It is widely used to induce diabetes in animal models. Administration of STZ in rodents via the intracerebroventricular (iv) route reduces cognition and promotes the formation of Aβ aggregates and phosphorylation of tau protein (Table 1) [77]. Due to its chemical nature, it can generate intracellular free radicals, nitric oxide (NO), hydrogen peroxide (H_2_O_2_), and mitochondrial damage. In addition, this compound causes neurodegeneration, oxidative and nitrative stress, glucose hypometabolism, a cerebral state of insulin resistance, progressive cholinergic deficiency [78], and neuroinflammation [79]. Similar biochemical, physiological, and anatomical effects are observed in patients with AD, making STZ a valuable means of obtaining animal models of AD. Models can be generated employing rats or mice. The rat model is obtained by administering 3 mg/kg of STZ under anesthesia; the injection is performed with the aid of a stereotaxic apparatus using the coordinates 0.9 mm posterior to the bregma point, ±1.8 mm lateral, and 3.8 mm vertical. The STZ is dissolved in 2 µL of artificial cerebrospinal fluid or sodium citrate buffer solution and is applied with a microsyringe. Administration can be performed on one or two occasions. The evaluation of the model is carried out three weeks after the injection [78,80].

In a mouse model, 3 mg/kg of STZ is administered unilaterally or bilaterally under anesthesia using a stereotaxic device with the coordinates −0.3 mm posterior, −1 mm lateral, and −2.5 mm vertical. As in the previous case, the STZ is dissolved and applied with a microsyringe. The model evaluation tests are carried out three weeks after the injection [81,82,83].

STZ-induced AD models have been evaluated and characterized for over 20 years, but their study has not yet been completed. Among the advantages that these models have over the transgenic animal model are that genetic manipulation does not begin at birth; pathological alterations can start at any age of the animal; the onset, development, and progression of cognitive impairments, as well as biochemical and structural changes, can be followed at different time points, and these models are suitable for studying potential drugs for prevention and treatment in AD [78].

**Table 1:** Characteristics of non-transgenic animal models of AD.

Model	Animal	Aβ	P-Tau	Neuro-Inflammation	Other Changes	Time	Selected References
Stretptozotocyn	Mouse Rat	✔	✔	✔	Oxidative and nitrative stress, glucose hypometabolism cerebral state of insulin resistance, progressive cholinergic deficiency, increased exploratory activity, impaired learning and spatial memory.	3 weeks	[81]
Scopolamine	Mouse Rat	✔	✔	✔	Neuronal apoptosis, changes in the expression of choline acetyltransferase (ChAT), acetylcholinesterase (AChE), and acetylcholine (ACh) receptors. Changes in memory evaluated by MWM, passive avoidance.	1 to 6 weeks	[84]
Aging	Mouse	✔	✔	✔	Oxidative stress, cognitive deficit, especially in spatial memory and learning tasks.	7–8 months	[85]
Mechanical stress	Mouse Rat	✔	✔	✔	Cognitive dysfunction, including impairments in memory and object recognition, along with behavioral disturbances such as anxiety and symptoms resembling depression.	1 month	[86]
Aluminum	Rat	✔		✔	Lipoperoxidation increased malondialdehyde levels, lower superoxide dismutase activity, and catalase activity.	4–6 weeks	[87]
Dietary patterns High-fat diet	Rat Mice	✔	✔	✔	Dysregulations in autophagy, apoptosis. Oxidative stress and decreases in long-term potentiation.	8–30 weeks	[88]

Both rat and mouse models have been used to study AD pathology and evaluate new pharmacological treatments. Assessed drugs include inhibitors of the enzyme choline acetylcholinesterase, NMDA receptor antagonists, antioxidants, anti-inflammatory drugs, antidiabetic compounds, glycogen synthase kinase 3β (GSK3β) inhibitors, and antihypertensives [78].

#### 3.4.2. Models Induced by the Administration of Scopolamine

The pathogenic mechanisms induced by scopolamine administration to mice or rats correlate with altered cholinergic function, increased oxidative stress, the induction of the amyloid cascade, and the expression of inflammatory mediators [89]. The mentioned changes contribute to the loss of cholinergic neurons and deficits in attention and memory (Table 1) [90]. In addition, scopolamine increased phosphorylated tau protein levels by >2-fold [91], thus highlighting the importance of post-translational phosphorylation modification in exacerbating tau pathology—increased hyperphosphorylated tau results as a consequence of the upregulation of GSK3β [91]. GSK3β, a tau kinase, may also be involved in AD pathogenesis through the upregulation of Aβ production and suppression of ACh synthesis [92].

Models can be generated employing rats or mice. Male 8–10-week-old mice weighing 25–30 g are used. After acclimation, animals are administered scopolamine at 0.75 mg/kg intraperitoneally (ip) once daily for seven consecutive days to induce memory impairment. The assessment of pathological changes occurs after treatment ends [93]. The Morris water maze test (MWM) evaluates the memory impairment produced by scopolamine administration in mice. Histological studies have demonstrated that in the CA1 region of the hippocampus, using the Nissl test, inflamed and vacuolated neurons show nuclear and apoptotic pyknosis, along with decreased neurons in the scopolamine model [94].

Additionally, changes in the expression of choline acetyltransferase (ChAT), acetylcholinesterase (AChE), and ACh receptors in the brains of rats that receive scopolamine have been reported [70]. A second model in mice involves administering scopolamine (4 mg/kg) via ip daily for 6 days. In this study, memory changes were assessed through the MWM test, and the levels of ACh and AChE were confirmed [95]. In addition, Wistar albino rats weighing 250 to 300 g can induce the scopolamine model. Scopolamine 1 mg/kg/ip is administered for 14 to 45 days. Once the treatment is finished, the evaluation tests are carried out [96]. The model is assessed by the passive avoidance memory test consisting of a dark/light two-compartment shuttle box separated by a door, with a conductor available on the floor of the dark compartment for electric shocks [97]. The object recognition test investigates short-term recognition memory functions [98]. Biochemical tests for reduced glutathione (GSH), malondialdehyde (MDA), and myeloperoxidase (MPO) are used to determine levels of oxidative stress [99].

#### 3.4.3. Models Induced by Aging

Aging is the most significant risk factor for SAD. The mechanisms underlying aging-related susceptibility to SAD are not well known. There are transgenic and non-transgenic rodent aging models in which animals are observed throughout their lives to study aging-related changes and underlying disease mechanisms and to test possible treatments [100]. One of the non-transgenic rodent models includes the following:

SAMP8 mice were developed from senescence-accelerated mice (SAM), which are senescence-accelerated strains that developed spontaneously from breeding pairs of the AKR/J series [85,101]. SAM are classified into two groups: senescence-accelerated prone mice (SAMP1, 2, 3, 6, 7, 8, 9, 10, and 11) and senescence-accelerated resistant mice (SAMR1, 4, and 5) [102]. SAMR strains show typical aging features, whereas SAMP strains show senescence acceleration and age-related pathological phenotypes similar to aging disorders observed in humans. SAMP mice display various clinical features of aging disorders as early as 7 or 8 months of age, which is approximately equivalent to middle age for mice compared to normal-aged mice (>20 months) [103]. Although they are not exact models of human aging, they exhibit aging-related physiological and, particularly, behavioral changes more rapidly than other mouse strains. They have been widely used in several studies of age-related neurodegeneration, including SAD [102,104].

The most important features of this model are as follows:

1. Premature aging with an early onset of age-related markers [102,103].
2. Memory and learning problems. One of the most studied traits of SAMP8 mice is their cognitive deficit, especially in spatial memory and learning tasks as they age [103,104,105].
3. Histopathological brain features similar to SAD in humans. As SAMP8 mice age, they accumulate Aβ plaques and neurofibrillary tangles in the brain, two hallmark histopathological AD findings in humans [104,105].
4. Oxidative stress. SAMP8 mice have been shown to have increased levels of oxidative stress in the brain, which contributes to cellular damage and may be related to their accelerated aging and the onset of neurodegenerative diseases [85,106].

These characteristics of SAMP8 mice make this model one of the leading models for studying the mechanisms of AD and testing potential treatments and therapies that may delay or mitigate the effects of aging and dementia (Table 1).

#### 3.4.4. Models Induced by Mechanical Stress

Many factors can alter the brain cells. Among the stressors that cause damage to the brain are electromagnetic radiation, temperature, and atmospheric pressure. These factors cause an essential change in the structural conformation of neuroglial proteins. Brain cells are sensitive to physical and mechanical stress. Indeed, various studies associate the development of neurodegenerative pathologies with exposure to injuries caused by traumas related to physical or work activity, as is the case of impact sports athletes, such as boxers and football players, where these patients present pathologies similar to those existing in AD [107]. The use of animal models enables one to illustrate that traumatic brain injuries can trigger amyloidogenic processing of APP and accumulation of Aβ in rodents. Tau pathology can be observed in rodent models subjected to mild repetitive trauma (Table 1). Although they may be nonspecific, neuroinflammation and neuronal loss can also be induced in these models through methods such as controlled cortical impact, weight drop impact, and repetitive mild trauma. Additionally, cognitive dysfunction—including memory impairment, object recognition issues, behavioral disturbances (such as anxiety and depression-like symptoms), and sensorimotor deficits—has been reported in models of TBI [108].

In patients with traumatic brain injuries, there is often an increase in the amount of Aβ at the injury site shortly after the trauma occurs. Similarly, studies on TBI conducted in mice have also revealed the presence of Aβ at the site of the injury [109]. Research has shown that specific proteins can move between interconnected cells via axons in different brain areas. This movement may play a crucial role in the development of amyloid pathology in AD brains [110]. Adult male BALB/c mice of approximately 6 weeks are commonly used to induce the model, weighing 19 and 24 g. Each mouse is exposed to repetitive sub-concussive impacts with stainless steel masses (marbles) of 13.5 or 20 g dropped at a height of 20 or 40 cm through a hollow tube to obtain impact forces on the dorsal face of the skull between 0.03 and 0.04 at 20 cm and between 0.04 and 0.08 J at 40 cm; the impact frequencies were one, two, or up to four per day at specific times for each group of study mice. The mice did not present neurological problems after the impacts; only those in which a force of 0.08 J was used presented some lethargy and some social isolation; the measurement of Aβ and tau protein was performed by confirmation with immunoassays; significant values were found in the studies carried out at higher altitudes, and at lower altitude, these values were lower. It is concluded that the best results were obtained with greater exposure to the processes and that this procedure is the most appropriate compared to other studies [111].

#### 3.4.5. Models Induced by Metals

In living organisms, metal ions are essential in trace amounts but can be toxic at high levels. Increased metal concentration in the brain has been linked to generating ROS, which can affect DNA and proteins and constitute part of the etiology of neurodegenerative diseases in aging. The accumulation of free metal ions such as iron (Fe^2+^), copper (Cu^2+^), zinc (Zn^2+^) [112,113], aluminum (Al^3+^) [114], and manganese (Mn^2+^), among others, has been correlated with AD, PD, amyotrophic lateral sclerosis, Wilson’s disease, hereditary ferritinopathy, and stroke [115].

Al^3+^ is not an essential element for living organisms, but it plays a role in several important processes. These include axonal transport, neurotransmitter synthesis, synaptic transmission, protein phosphorylation and dephosphorylation, protein degradation, gene expression, and the inflammatory response. However, high concentrations of aluminum in the brain can lead to spatial memory deficiencies, affect emotional reactions, and impair various brain functions related to memory and learning. [116]. The toxicity mechanism of Al^3+^ is not fully known. However, it has been described that due to its chemical nature, it has an affinity for negatively charged molecules such as phosphate, carboxyl, and hydroxyl groups of biomolecules, with which it can establish covalent bonds and affect their topology; when binding to DNA, it modifies gene expression [117]. When binding to ATP, it influences energy metabolism; this ion also inhibits the function of protein phosphatases and protein kinases, as well as ion-activated and exchanger proteins [116], inhibits dephosphorylation of the tau protein, and increases its aggregation [118]; in addition, it promotes the oxidation of Fe, increases the formation of ROS, and causes an inflammatory response in different tissues [114]. In the past decade, it was reported that rats treated with chronic intake of Al^3+^ showed a significant increase in the expression of the APP gene in the cerebral cortex and hippocampus, suggesting that one of the mechanisms contributing to the neuropathological picture of Al^3+^ in AD is through the activation of the cascade of formation of the Aβ peptide [119]. In recent decades, AlCl_3_ has been used to generate rat models that reproduce (albeit partially) the symptoms and pathophysiological alterations of AD.

According to bibliographic findings, there are two processes for generating rat models with AD through the administration of AlCl_3_. The first consists of randomly selecting Wistar rats weighing 230 ± 20 g and administering 4.2–50 mg/kg of AlCl_3_ dissolved in water ip for 28–42 days (depending on the experimental design); 24 h later, behavioral assessment tests, including the MWM, are performed, and 24 h later, biochemical tests of MDA levels, superoxide dismutase activity, and catalase activity) [120,121] are carried out to screen individuals that mimic the symptomatology and neuropathology of AD. The second consists of randomly selecting Wistar rats weighing 100–200 g and administering a combination of AlCl_3_ and d-galactose (d-gal), in the amounts of 150 mg/kg and 300 mg/kg, respectively, dissolved in saline solution (0.9%), for one week. Twenty-four hours later, the behavior is evaluated employing MWM, and 24 h later, they are sacrificed. To assess the model, the corresponding biochemical and neurochemical tests are performed: lipoperoxidation, MDA levels, superoxide dismutase activity, and catalase activity) [122,123]. Both processes give rise to animals that reproduce the symptoms of AD and are suitable for studies of the mechanisms involved in it. The main advantage of the two methods is that the latter requires less experimental time and a lower cost [124]. Rat models of induced AD have been used in the evaluation of novel neuroprotective compounds for the prevention and future treatments of AD in humans [125].

#### 3.4.6. Dietary Patterns

Dietary patterns have been examined as modifiable risk factors for cognitive decline, dementia, and related conditions [126]. Clinical studies have explored various dietary patterns, including the Mediterranean diet, ketogenic diet, and DASH diet, and their effects on AD pathology. These studies suggest that a diet low in sugar and high in omega-3 polyunsaturated fatty acids and antioxidant compounds may be beneficial in preventing cognitive decline [127]. Conversely, adhering to an unhealthy diet can increase the risk of AD. In a cross-sectional study, a preference for junk food significantly predicted cerebral Aβ deposition [128].

Additionally, the consumption of a high-fat diet (HFD) is a major contributor to overweightness and obesity. This can lead to elevated levels of circulating free fatty acids, resulting in low-grade inflammation due to the increased release of proinflammatory cytokines. These inflammatory processes activate signaling pathways, including serine kinases, IKKβ, and JNK1, which inhibit insulin receptor signaling and enhance insulin resistance. This situation contributes to cognitive impairment and neurodegeneration [129]. Furthermore, high fat and high fructose consumption are associated with diabetes and hyperinsulinemia, both of which are risk factors for developing SAD. A link between obesity and AD has been suggested, where insulin resistance plays a role in regulating inflammatory processes, adipokine dysregulation, oxidative stress, and mitochondrial dysfunction [130].

Non-transgenic animal models have proven useful for studying various dietary patterns’ molecular and cellular basis and their relationship with SAD [131]. For instance, Busquets et al. reported the formation of Aβ in C57BL/6J mice fed an HFD over a long period. These mice also exhibited dysregulations in autophagy and apoptosis alongside exacerbated inflammatory processes [132]. The HFD induces brain insulin resistance by reducing the tyrosine phosphorylation of the insulin receptor and increasing the serine phosphorylation of insulin receptor substrate 1, which can disrupt the proteins involved in the insulin signaling pathway [131]. Additionally, an HFD exacerbates neuroinflammation and oxidative stress while diminishing long-term potentiation in the hippocampus [132].

However, this model has limitations related to the varying compositions of diets reported and the different time points used to evaluate molecular pathways. For example, studies involving an 8-week HFD consumption period have indicated BBB damage associated with microglial activation, increased oxidative stress, elevated proinflammatory cytokines, and memory impairment [133]. In contrast, consuming an HFD for 30 weeks increases protein expression of phosphorylated tau, APP, and BACE1 cleaving enzymes, leading to the formation of Aβ plaques and neurofibrillary tangles [134]. Conversely, short-term consumption periods of an HFD (3, 7, or 10 days) affect several markers of inflammation, endoplasmic reticulum stress, and apoptosis in the hippocampus of young mice [135]. Therefore, the molecular and cellular changes over time in this model remain incompletely understood and may offer temporally relevant biological insights.

## 4. Discussion

Animal models of AD have played a crucial role in defining critical disease-related mechanisms and are essential for evaluating novel therapeutic approaches. Many treatments currently being tested in clinical trials originate from studies conducted on rodent models. The current transgenic models of AD are derived from familial FAD and are histopathologically indistinguishable from SAD [136].

However, SAD’s mechanisms are more complex and varied than the monogenic dominant mutations responsible for FAD, making them less understood and less extensively modeled [137]. Transgenic models that overproduce mutant APP lead to amyloid accumulation. Due to the complexity of the disease, none of the transgenic lines have been able to replicate all aspects of AD pathology. This highlights the limitations of using transgenic systems to reproduce the human disease process fully.

Doubly mutated transgenic models with increased Aβ accumulation develop lesions earlier [138]. In contrast, neurofibrillary tangles are primarily found in transgenic mice that overexpress mutated tau [139]. While mixed transgenic models that incorporate both Aβ and tau accumulation mimic the histological changes observed in AD, the genetic modifications required to express these pathological features differ from those found in familial FAD. Additionally, many transgenic AD models fail to replicate the human condition’s neuronal and synaptic loss, characteristic of the disease.

Furthermore, the inflammation present in these models does not accurately reflect the inflammation seen in humans with AD, as there are notable differences in the nature and severity of the inflammation between humans and AD transgenic mice [140]. The complexity of the disease suggests that no single transgenic line can replicate all aspects of AD pathology, highlighting the limitations of using transgenic systems to model human disease processes.

Another concern with transgenic AD models is that the overproduction of Aβ drives the pathological process in line with the amyloid cascade hypothesis. These models effectively reproduce FAD, while the majority of individuals experience SAD. In SAD, the disease is linked to an imbalance between the production and clearance of Aβ, leading to its accumulation over time. However, the mechanisms behind this imbalance remain poorly understood [140].

From a practical perspective, there is often a discrepancy between preclinical animal models and human clinical trial results. While transgenic models enhance our understanding of AD pathogenesis, their limitations highlight the need to explore new approaches in AD animal modeling.

In contrast, non-transgenic experimental models of AD serve as a valuable and complementary preclinical strategy alongside transgenic animal models. These non-transgenic approaches demonstrate the ability to resemble the key mechanisms underlying neurodegeneration in AD [141], including Aβ deposition, tau hyperphosphorylation and neuroinflammation (Table 1). Furthermore, it is imperative to study the combination of various non-transgenic models to evaluate changes in Aβ deposition, tau protein phosphorylation, neuroinflammation, and subsequent cognitive impairment. This is because patients with AD generally present several pathophysiological alterations that converge toward the development of the disease [3]. This would facilitate understanding of the changes that promote its development, in addition to providing a model that allows for the evaluation of known and novel therapeutics, particularly those that have shown favorable outcomes in this type of model. This standardization should involve using a single animal model and assessing the same therapeutic strategy across different models. Utilizing non-transgenic animal models facilitates the investigation of risk factors and the identification of molecular mechanisms contributing to SAD. This research domain is currently underexplored but holds the potential for advancing the early identification of individuals at risk and the development of innovative therapeutic strategies. While the present landscape of preclinical research examining therapeutic interventions in non-transgenic animal models of SAD remains limited and incomplete, we advocate that these models may enhance drug development. They offer a cost-effective, accessible, and efficient research platform, allowing for a shift in the focus of drug development from traditional amyloid-targeted strategies to novel pathological approaches within the context of AD.

## 5. Conclusions

AD is a devastating condition that significantly impacts society. Despite decades of research and billions of dollars invested, effective treatments have not yet reached the market. Most drug candidates that have failed in clinical trials showed anti-AD activity in various transgenic animal models. While transgenic mouse models have been the primary method used in drug development research to evaluate potential therapies, it is crucial to recognize the need for animal models that better reflect the key pathological changes associated with similar risk factors.

These models could play an increasingly important role in current drug development efforts for AD. Therefore, there is an urgent need for a model that accurately represents the changes seen in sporadic SAD, the most common form of AD, which could facilitate successful drug development in the future.

## Abbreviations

The following abbreviations are used in this manuscript:

α7 nAChR	α7-nicotinic acetylcholine receptor
ACh	acetylcholine
AChE	acetylcholinesterase
AD	Alzheimer’s disease
ChAT	cholineacetyltransferase
ATP	adenosine triphosphate
Al^3+^	aluminum
AD	Alzheimer’s disease
APP	amyloid β precursor protein
Aβ	amyloid-β
fAβ	amyloid-β fibril
oAβ	amyloid-β oligomer
ALS	amyotrophic lateral sclerosis
BACE1	β secretase
PrPc	cellular prion protein
CNS	central nervous system
CTE	chronic traumatic encephalopathy
Cu^2+^	copper
d-gal	d-galactose
FAD	familiar form of AD
RAGE	glycation product receptor
GSK3β	glycogen synthase kinase 3β
H_2_O_2_	hydrogen peroxide
HFD	high-fat diet
·OH	hydroxyl radical
P-Tau	hyperphosphorylated tau
HIF-1α	hypoxia-inducible factor 1-alpha
IGF	insulin-like growth factor
icv	intracerebroventricular
ip	intraperitoneal
Fe^2+^	iron
MDA	malondialdehyde
MCI	mild cognitive impairment
Mn^2+^	manganese
MWM	Morris water maze
MPO	myeloperoxidase
NMDA	*N*-methyl-d-aspartate
NFTs	neurofibrillary tangles
NO	nitric oxide
PD	Parkinson’s disease
PSEN1	Presenilin 1
PSEN2	Presenilin 2
P-tau	hyperphosphorylated tau
ROS	reactive oxygen species
GSH	reduced glutathione
SAM	senescence-accelerated mouse
SAD	sporadic form of AD
STZ	streptozotocin
TBI	traumatic brain injury
WD	Wilson’s disease
Zn^2+^	zinc
